# Quantification of nitromethane in mainstream smoke using gas chromatography and tandem mass spectrometry

**DOI:** 10.1016/j.toxrep.2021.02.008

**Published:** 2021-02-15

**Authors:** Juliana Giraldo Junco, Gala M. Chapman, Roberto Bravo Cardenas, Clifford H. Watson, Liza Valentín-Blasini

**Affiliations:** Centers for Disease Control and Prevention, 4770 Buford Highway NE, Atlanta, GA, 30341, United States

**Keywords:** Nitromethane, Mainstream smoke, GC–MS/MS, Tobacco analysis

## Abstract

Nitromethane is a volatile organic compound categorized as a Group 2B carcinogen by the International Agency for Research on Cancer. It has been detected in mainstream cigarette smoke, but few reliable methods have been reported for accurate quantification. We developed, a sensitive, selective, fully validated method for the targeted determination of nitromethane in mainstream tobacco smoke in ten U.S. domestic brands and two quality control materials (3R4F and CM6). The vapor phase portion of machine-generated cigarette mainstream smoke, under modified ISO 3308:2000 regime (ISO) and modified intense regime (HCI), from single cigarettes was collected using airtight polyvinylfluoride sampling bags. The bags’ contents were extracted using methanol containing an isotopically labeled internal standard followed by gas chromatography-tandem mass spectrometry. This approach is sufficiently sensitive to measure nitromethane levels in the nanogram range, with a method limit of detection of 72.3 ng/cig. Within-product variability estimated from the replicate analysis of 10 products ranged from 4.6%–16.3% (n = 6) over the two different smoking regimes, and method reproducibility estimated from two products used as quality control materials (3R4F and CM6) yielded intermediate precision values ranging from 16.6 to 20.8% (n = 20). Under HCI, nitromethane yields in machine-generated cigarette smoke from ten different domestic cigarette products ranged from 3.2 to 12 μg/cig; under ISO yields ranged from 1.6 to 4.9 μg/cig under standardized smoking machine conditions.

Nitromethane yields are related to both the smoke regime (blocking of vent holes, puff duration and puff volume) and the heterogeneity of tobacco mixtures. This method provides a selective and fully validated technique to accurately quantify nitromethane in mainstream cigarette smoke, with minimal waste generation. It is an improvement over previous methods with regards to specificity, throughput, and simplicity of the sample collection process.

## Highlights

• A novel method was developed to detect nitromethane in cigarette smoke.
• The method is sensitive, accurate, and fit for high-throughput applications.
• The new method was used to quantify nitromethane in smoke from 10 cigarette brands.
• Differences were observed depending on tobacco types used.

## Graphical abstract

**Figure:**
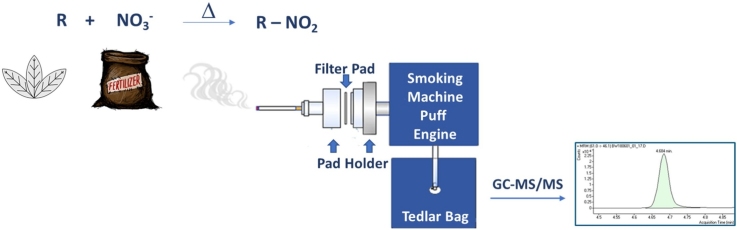

## 1 Introduction

Mainstream cigarette smoke contains more than 7000 different chemicals [[1], [2], [3], [4], [5], [6], [7], [8], [9], [10], [11]]; the U.S. Food and Drug Administration (FDA) has identified many of those chemicals as harmful or potentially harmful constituents (HPHCs) [12]. The toxicity and carcinogenicity of the chemicals in cigarette smoke have made smoking a leading cause of preventable and premature deaths in the United States. Smoking is linked to increased risks for cardiovascular disease, chronic obstructive pulmonary disease, and many types of cancer [[13], [14], [15], [16], [17], [18]]. Nitromethane is one of the toxic nitro compounds included in the FDA’s HPHCs list because of its probable carcinogenicity, as indicated in animal, exposure, metabolic, and structure-activity relationship studies [19,20]. Nitromethane forms from C–H radicals and nitrogen oxides during combustion of the tobacco during smoking. Accurate quantitation of this compound in cigarette smoke is important for estimating potential exposure risks [21].

Quantification of nitromethane in cigarette smoke is challenging because of nitromethane’s volatility and low molecular weight. Because of the complexity of the tobacco smoke matrix, nitromethane often co-elutes with chromatographic interferents [22]. Accordingly, relatively few experimental methods have been developed to accurately quantify nitromethane in mainstream tobacco smoke.

In 1968, Hoffmann and Rathkamp were the first to report nitromethane levels in cigarette smoke. Their approach involved collecting the smoke of 20–200 cigarettes in water-filled impingers. They subjected the samples to a lengthy and laborious series of distillations and liquid-liquid extractions before using gas chromatography with an electron capture detector (GC-ECD) in a multi-analyte method for analysis [23].

More recently, Sampson et al., developed a multi-analyte volatile organic compound (VOC) panel that includes nitromethane. In that study, VOCs derived from cigarette smoke were collected in Tedlar bags and concentrated using solid phase microextraction before analysis using gas chromatography–mass spectrometry (GC–MS) [24]. The method is suitable for a broad range of VOCs, improves throughput relative to prior approaches, and eliminates VOC breakthrough concerns related to impinger trapping. However, the chromatographic interferences limited the accuracy for several analytes, including nitromethane.

In 2015, Wang et al. published an analytical method for determining nitro compounds in mainstream cigarette smoke. They collected smoke from 20 cigarettes, using two consecutive impingers filled with ethyl acetate, then added 2-methyl-2-nitropropane as an internal standard. Sample separation and quantification were carried out using heart-cutting multidimensional gas chromatography and mass spectrometry (GC-GC–MS) [25]. Although this approach is useful for reducing co-eluted interferences, the sample collection and chromatography are time consuming and complicated. Wang et al. did have a few limitations such as not reporting an assessment of matrix interferences and using a surrogate internal standard rather than an isotopically labeled nitromethane standard.

Because of the challenges inherent in quantitation of this volatile nitro compound, a targeted method was developed for accurately measuring nitromethane in mainstream cigarette smoke. This new method is fully validated, sensitive, selective, accurate, and suitable for high-throughput applications. Nitromethane levels were assessed in mainstream cigarette smoke using a simple “extract and shoot” approach requiring smoke collection from a single cigarette. An isotopically labeled internal standard (nitromethane-*d*_3_) was selected to reduce the probability of matrix effects and account for any potential sample instability. Sample separation was carried out using gas chromatography (GC) and quantitation was carried out using tandem mass spectrometry (MS/MS). This detection technique promotes the selective and sensitive detection of the low molecular weight analyte without necessitating laborious sample cleanup or a long chromatographic separation time.

## 2 Experimental

### 2.1 Chemicals and materials

Nitromethane (CAS 75-52-5) standards were purchased from SPEX Certiprep (Metuchen, NJ, USA) as a certified reference material, 1000 μg/mL solution in methanol. High-performance liquid chromatography grade methanol (CAS 67-56-1) was purchased from Fisher Scientific. Deuterated internal standard nitromethane-*d*_3_ (CAS 13031-32-8) was purchased from O2Si Smart Solutions (Charleston, SC, USA) at a concentration of 5000 mg/mL in methylene chloride. Nitromethane certified reference materials and internal standards were stored at −70 °C when not in use. 1 L Tedlar® Push Lock Valve (PLV) gas sampling bags with Thermogreen® LB-2 septa were purchased from Sigma-Aldrich (St. Louis, MO, USA).

Ten different popular American cigarette products were acquired in the metropolitan Atlanta area through The Lab Depot, Inc. (Dawsonville, GA, USA). The selection represented four major domestic cigarette manufacturers: Philip Morris, ITG, R.J. Reynolds (three products each), and Liggett Group (one product). University of Kentucky 3R4F research cigarettes (Lexington, KY, USA) and CORESTA Monitor #6 (CM6) test-piece reference cigarettes were used as QC materials.

Cigarettes were kept in a −20 °C freezer until needed. Prior to smoking, cigarettes were conditioned for at least 48 h and no more than 10 days in a temperature-controlled and humidity-controlled smoking chamber (Parameter Generation & Control Inc., Black Mountain, NC, USA) at 22 ± 1 °C and 60 % ± 3 % relative humidity. Cambridge filter pads (CFPs) also were kept in the same chamber, in accord with ISO 3402:1999 conditioning and testing guidance and CORESTA recommended method No. 21 [26,27].

### 2.2 Smoking instrumentation

Cigarettes were smoked on a Cerulean SM450 20-port smoking machine (Cerulean, Richmond, VA, USA) using Cerulean industry-standard smoke holders (Molins PLC, Milton Keynes, UK) fitted with 44 mm Cambridge filter pads (Borgwaldt, Hamburg, Germany). Cigarette smoke collection bags were attached to the exhaust ports of the puff engines using 3.5-inch lengths of polyvinyl chloride tubing. A soap bubble meter from Borgwaldt (Hamburg, Germany) was used to verify smoking machine puff volumes prior to each smoking run. After collection of the mainstream smoke vapor phase, the bags were spiked with extraction solution containing internal standard and were shaken for 30 min on an Eberbach 6010 fixed speed, reciprocal shaker (Eberbach Corporation, Ann Arbor, MI, USA), 45 min after smoking was completed. Research cigarettes 3R4F and CM6 were smoked in parallel with the other samples and used as quality control materials; smoke runs were accepted or rejected in accordance with a modified set of Westgard QC rules [28].

### 2.3 Smoking conditions

Cigarettes were smoked to the filter overwrap plus 3 mm or to a butt length of 23 mm, whichever was longest. Each sample was obtained by smoking a single cigarette, following either a modified ISO 3308:2000 regime (35 mL puff volume, 2 s puff duration, and 60 s between puffs with filter ventilation) or a modified intense regime (HCI: 55 mL puff volume, 2 s puff duration, and 30 s between puffs with 100 % filter ventilation blockage). At the end of each smoking run, two clearing puffs were collected to flush any remaining smoke from the lines.

### 2.4 Analytical instrumentation

Nitromethane was quantitatively analyzed using an Agilent 7890B GC system coupled to an Agilent 7000C tandem mass spectrometer (GC–MS/MS) (Agilent Technologies, Santa Clara, CA, USA) equipped with a Gerstel MPS autosampler rail (GERSTEL GmbH & Co. KG, Mülheim an der Ruhr, Germany). The GC inlet was equipped with an ultra-inert universal gooseneck inlet liner with glass wool (Agilent 5190-3165), kept at 230 °C with a 30-psi injection, and the flow rate was maintained at 55.8 mL/min with a 60:1 split ratio. A 40 m Agilent DB-VRX Ultra Inert capillary column with a 180 μm internal diameter (I.D.) and a 1.0 μm film thickness was used for separation. Research-grade helium (Airgas, Inc., Radnor, PA, USA) was the carrier gas. The oven was temperature-programmed, beginning at 30 °C, progressing to 80 °C at a rate of 15 °C/min, then to 230 °C at a rate of 100 °C/min, and held at 230 °C for 5 min.

Mass spectral detection utilized electron ionization and multiple reaction monitoring (MRM). The source was heated at 230 °C with an ionization voltage (electron energy) of −40 eV, and both quadrupoles (MS1 and MS2) were heated to 150 °C. We used ultra-high purity grade nitrogen (Airgas) as the collision cell gas. Based on abundance, two MRM transitions were selected. The most abundant fragment transition from the molecular ion was used for quantitation, and the second most abundant fragment transition for confirmation. A third MRM transition was used for the internal standard. For all transitions, the MS1 and MS2 resolution was set to “wide,” with a dwell time of 60 ms. Table 1 shows all transition and collision energies used for the quantification. Agilent MassHunter Workstation software was used for data acquisition and quantification. Analyte concentrations were calculated from the ratio of native analyte peak area response to internal standard peak area response after inspection of peak symmetry, retention time, and quantitation and confirmation ion ratios.

**Table 1:** Ion transitions and collision energies.

Compound	Transition type	Transition ion masses (m/z)	Collision energy (V)
Nitromethane	Quantitation	61 → 46.1	7
	Confirmation	61 → 30.1	1
Nitromethane-*d_3_* (internal standard)	Quantitation	64.1 → 46.1	6

### 2.5 Preliminary screening for nitromethane in particulate matter, cigarette filter and vapor phase

Nitromethane recoveries were compared in the various smoke fractions obtained following the machine smoking of single cigarettes (CFP particulate phase, cigarette filter particulate phase, and Tedlar bag vapor phase). Smoke fraction nitromethane recoveries were compared in triplicate for three different commercial cigarette products and two research products (3R4F and CM6).

From each corresponding port, a 1 L Tedlar bag, a filter pad, and a cigarette filter were collected. Pads and cigarette filters were placed in separate 20 mL amber vials and sealed with polytetrafluoroethylene (PTFE) faced caps immediately following smoking. All samples were spiked with 10 mL of methanolic extraction solution containing isotopically labeled internal standard. The extraction solution was added into both the vials and the Tedlar bags using an Eppendorf Repeater Xstream repeating pipette fitted with a 10 mL Combitip (Eppendorf North America, Hauppauge, New York); extraction solution was injected into bags through the push-lock valve using the repeating pipettor. Bags and vials were shaken for 30 min at 180 rpm on a Barnstead Lab-line E-class orbital shaker (Dubuque, IA, USA). Extracts were analyzed by GC-MS/MS using the method described in the “Analytical Instrumentation” section.

### 2.6 Extraction method

Nitromethane was not detected in the preliminary analysis of CFPs and cigarette filters. For that reason, their analyses were discontinued, and only the contents of the Tedlar bags were included for the nitromethane analysis. After smoking, a 45-minute wait time allowed the gas-solid phase equilibrium to establish between the vapor phase of the mainstream smoke and the internal walls of the collection bag. Previous ruggedness testing of the analytical methods indicated that 45 min yielded a maximal recovery of nitromethane. After establishing equilibrium, 10 mL of methanolic extraction solution containing the internal standard was injected into the bag through the push-lock valve using a repeating pipette fitted with 10 mL Combitip. The bags were closed and manually shaken to ensure that all the nitromethane on the internal walls was rinsed with the extraction solution. Then bags were placed on a shaker at 180 rpm for 30 min to homogenize the nitromethane and the internal standard in the solvent. Methanol was chosen as the extraction solution over other solvents such as acetone, dichloromethane, and ethyl acetate, for its easier storage, better bag compatibility, and better solvation properties.

### 2.7 Standard preparation

Certified reference material nitromethane was used to prepare nine levels of calibrators within a range of 0.0200–3.00 μg/mL concentrations to cover values of nitromethane in mainstream cigarette smoke in different commercial cigarette products. Nitromethane ampoules were stored at −70 °C. Ampoules were brought to room temperature and vortexed before use to ensure a homogenized solution, then diluted with methanol to prepare spiking solutions. Standards were prepared in 1 L Tedlar bags filled partially with room air in order to better approximate cigarette sampling conditions. Spiking solution was injected into the sampling bag via the septum using a gas-tight syringe. After spiking, a 10 mL volume of extraction solution containing 1.5 μg/mL isotopically labeled nitromethane was injected into the bag via push-lock valve. Bags were shaken first by hand for a complete mixing of nitromethane and internal standard with the extraction solution and then shaken for 30 min at 180 rpm using a reciprocal shaker. Calibrators not intended for same-day use were stored in amber autosampler vials at −70 °C for up to 15 days. Calibrators were taken to room temperature and vortexed before use.

## 3 Results and discussion

### 3.1 Preliminary screening for nitromethane in particulate matter, cigarette filter, and vapor phase

**Fig. 1:**
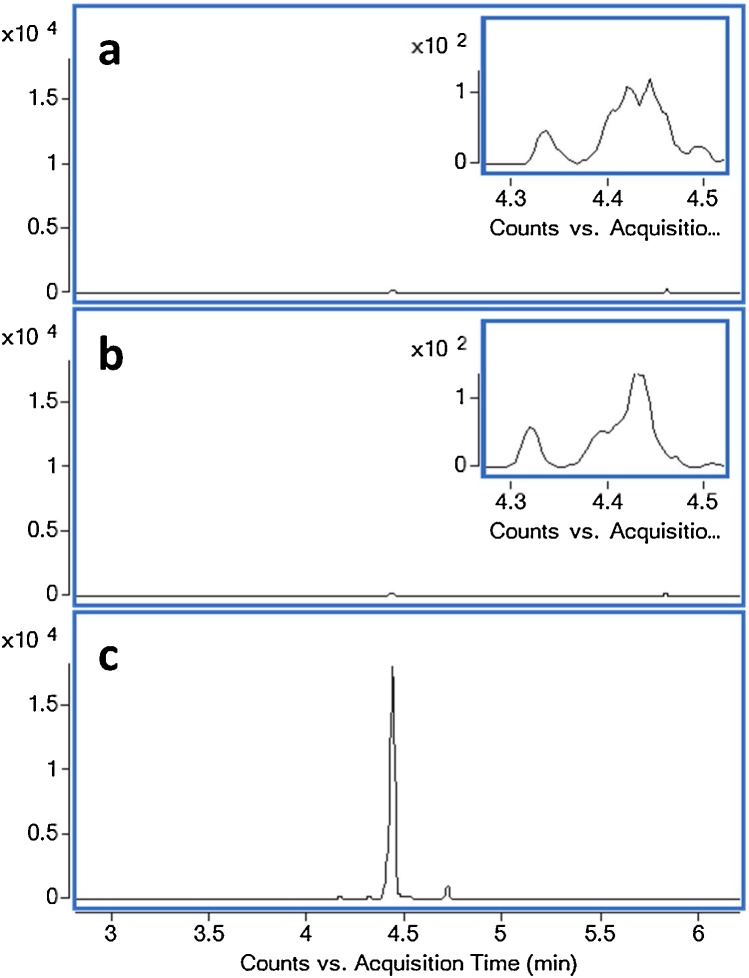
Representative chromatograms (confirmation transition, 61.0 → 30.1) illustrating relative nitromethane recoveries from (a) cigarette filter particulate matter, (b) CFP particulate matter, and (c) vapor phase collected in Tedlar bag from a commercial cigarette product. Inset: magnified region for nitromethane retention time in filter and pad illustrating no significant recovery signal.

For all of the products studied, nitromethane was detected only in the vapor phase (Tedlar bag) fractions; no detectable levels of nitromethane were observed in either the cigarette filter or CFP fractions. Accordingly, only the contribution of the vapor phase was considered for nitromethane quantitation in smoke samples. A comparison of chromatograms obtained for the bag, pad, and filter fractions of a representative cigarette product is provided in Fig. 1.

### 3.2 Method validation

#### 3.2.1 Precision

Intermediate method precision was evaluated by analyzing QC materials (3R4F-QC high and CM6-QC low) under both the ISO and HCI smoking regimes (n = 20 per regime). Smoke runs were performed over the course of 20 different days; each data point for each QC material represents a different smoke run. The resulting relative standard deviations for 3R4F were 17.9 % and 18.8 % using the HCI and ISO regimes, respectively; the resulting relative standard deviations for CM6 were 16.6 % and 20.8 % using the HCI and ISO smoke regimes, respectively. Product variability estimates calculated from the analysis of commercial products (n = 6) yielded standard deviations ranging from 7.0–16.3 % for the HCI regime and from 4.6–14.4 % for the ISO regime.

#### 3.2.2 Accuracy

Method accuracy was assessed by spiking CM6 smoke samples with low, medium, and high concentrations of nitromethane spiking solution and calculating their respective percent recoveries relative to the calibration curve. Accuracies were assessed in triplicate for each spike level. The average calculated percent recoveries for ISO regime samples were 99.8 %, 96.4 %, and 98.1 % for low, medium, and high spike levels, respectively. Standard deviation was no greater than 3.5 %. The same was done with HCI regime samples, which gave values of 97.8 %, 99.8 %, and 98.4 % for low, medium, and high spike levels, respectively. Standard deviation did not surpass 2.9 %. All values were deemed acceptable.

#### 3.2.3 Limit of detection

Limits of detection (LOD) were calculated by Taylor’s method^18^ and from evaluating the signal-to-noise ratio, S/N value of 3, of a low concentration nitromethane solution (0.02 μg/mL) injected during 10 different days over a 42 days lapse, based on the mean S/N value (quantitation transition; peak-to-peak noise) of the 10 measurements. The latter approach yielded a higher estimated LOD of 7.23 ng/mL (72.3 ng/cig); thus, we report the higher LOD as the more conservative approach.

#### 3.2.4 Ruggedness

To optimize the method’s efficiency, ruggedness studies were performed testing key parameters. Tests were performed with zero, one, and two clearing puffs. The results indicated no significant difference in nitromethane recovery relative to the method value (1 run clearing puff) when this parameter was varied. We also assessed the time before extraction (i.e. time between smoking completion and introduction of extraction solution to Tedlar bags; intervals ranging from 15 to 75 min were tested). Better results were achieved when samples were extracted 45 min after smoking. While the reason for this result is not clear, it could potentially be attributed to a gas-phase/solid-phase equilibrium in the collection bag facilitating extraction and recovery of the analyte following solvent introduction.

Changes in extraction time (sample residence time on the reciprocal shaker) were evaluated; extraction times of 15, 30, and 60 min were tested. The results indicated that maximal recoveries were obtained at 30 min. Assessment of extraction volume (varied from 4−12 mL) indicated that recoveries were optimal with 10 mL of extraction solution. Chromatographic settings were tested too. Small changes in inlet temperature showed significant changes in nitromethane signal. Inlet temperature affects nitromethane and internal standard peak areas; both increase with increasing inlet temperature. The effect of using CFPs from different manufacturers was tested, but no significant difference in nitromethane recovery was observed on comparison of Borgwaldt and Whatman (Whatman, Pittsburg, PA) brand CFPs. Tedlar gas sampling bags from different manufacturers were evaluated; no significant difference in nitromethane recovery was observed on comparison of bags obtained from Supelco with those obtained from Environmental Sampling Supply (San Leandro, CA).

#### 3.2.5 Stability

To assess thermal stability and photostability of nitromethane in standards and smoke extract samples, low and high concentration calibrators and smoke matrix samples were tested under three different conditions. One group of standards and samples was kept at room temperature (20 °C) with light exposure. Two other groups were kept in the dark, one at room temperature (20 °C), the other in the freezer (−70 °C). To eliminate the likelihood of internal standard degradation from relative breakdown, internal standard ampoules were kept unopened in the freezer until the day of measurement. Each day of analysis, a single ampoule of internal standard and an ampoule of each of the different samples from each environment were equilibrated to room temperature, then spiked with internal standard solution, vortexed and analyzed. Based on percent changes relative to the original sample responses, smoke samples and calibrators are stable for up to 17 days when stored at −70 °C; over this time period, percent changes of 2.7 and 3.7 % were observed for the low and high standards, respectively, and a 6.2 % change was observed for the smoke matrix sample. Exposure to light (and to a lesser extent, higher temperatures) increased the rate of apparent change in concentration for smoke samples (but not calibrators) within this time period. In accordance with these results, calibrators were always used within 14 days of being prepared.

#### 3.2.6 Matrix effects

Matrix effects can have a significant impact on quantitation when calibrators are prepared in a different matrix than samples, and these effects can be particularly significant when the internal standard is structurally distinct from the target analyte. Despite the application of a deuterated nitromethane internal standard, matrix effects measurements were nonetheless a necessary component of method validation given the use of solvent-based calibrators. Matrix effects were assessed comparing the slopes of ten-point calibration curves prepared in either smoke vapor (matrix) extracts or non-matrix (blank methanol) extraction solution, equivalent to smoke samples and calibrators, respectively. Least squares slopes were calculated for three independent calibration curves, averaged for the matrix-based and non-matrix-based samples. The averaged slopes were compared for both sample sets. Both matrix-based and non-matrix-based calibrators had acceptable linearity (R^2^ ≥ 0.99). Matrix effects were minimal, with an average difference of 2.26 % between slopes for the ISO regime and 0.73 % for the HCI regime.

### 3.3 Analysis of cigarette products

The smoke from 10 different cigarette products representing four major cigarette product manufacturers (R.J. Reynolds, Philip Morris, Liggett Group, and ITG) were analyzed, all together with two research cigarette products (University of Kentucky 3R4F reference cigarettes and CORESTA Monitor #6 (CM6) test pieces). The smoke was analyzed for nitromethane content (n = 6) using two different smoke regimes (HCI and ISO). Table 2, Table 3 show the results of this analysis.

**Table 2:** Nitromethane results (μg/cig) for cigarette brands smoked under HCI modified regime.

Product No.	Average (μg/cig)	Standard Deviation (μg/cig)	% RSD	Manufacturer
**1**	3.2	0.5	16.3	R.J. Reynolds
**2**	12	1.8	14.7	R. J. Reynolds
**3**	11.6	0.8	7.0	R.J. Reynolds
**4**	12	1.4	11.5	Philip Morris
**5**	11.3	1.4	12.3	Philip Morris
**6**	8.7	1.1	12.6	Philip Morris
**7**	11.3	1.6	14.1	ITG
**8**	11.8	1.6	13.2	ITG
**9**	8.4	1.1	13.2	ITG
**10**	9.1	0.9	9.4	Liggett Group
**11**	9.4	1.0	10.3	University of Kentucky
**12**	3.5	0.4	12.5	CORESTA

**Table 3:** Nitromethane results (μg/cig) for cigarette brands smoked under ISO modified regime.

Product No.	Average (μg/cig)	Standard Deviation (μg/cig)	% RSD	Manufacturer
**1**	1.6	0.1	8.9	R.J. Reynolds
**2**	4.8	0.4	8.5	R. J. Reynolds
**3**	3.8	0.3	7.9	R.J. Reynolds
**4**	4.1	0.4	9.3	Philip Morris
**5**	4.4	0.3	7	Philip Morris
**6**	3.4	0.3	8.7	Philip Morris
**7**	4.3	0.2	4.6	ITG
**8**	4.9	0.6	12.6	ITG
**9**	2.9	0.2	7.3	ITG
**10**	3.7	0.5	14.4	Liggett Group
**11**	2.7	0.3	11.3	University of Kentucky
**12**	1.7	0.2	9.8	CORESTA

The lowest yields of nitromethane were observed for the two Virginia blend products analyzed: deliveries were 1.6 and 1.8 μg/cig for the ISO regime and 3.2 and 3.6 μg/cig for the HCI regime. The other products analyzed were American blends, which demonstrated significantly higher levels of nitromethane: deliveries ranged from 2.7 to 4.9 μg/cig for the ISO regime and 8.4–12 μg/cig for the HCI regime. Nitro compounds are synthesized during tobacco combustion via reactions between hydrocarbon radicals and nitrates; accordingly, these results are as expected given the differences in nitrate content in the tobacco blends: Virginia blend cigarettes contain only low-nitrate Virginia tobacco, whereas American blend cigarettes contain high-nitrate Burley tobacco as part of a mixture [[29], [30], [31]].

The range of variability observed in the results (exhibiting relative standard deviations of 4.6–16% for both smoke regimes) can be attributed at least in part to the inherent heterogeneity of tobacco mixtures. In this method, each sample represents only a single cigarette; accordingly, higher variabilities are to be expected relative to methods utilizing multiple cigarettes per sample. Variability in deliveries may also be partially attributable to the volatility of nitromethane and environmental factors during the sample collection process.

Yields obtained using this method for 3R4F reference cigarettes were slightly higher than those previously reported in the literature [[12], [13], [14]]. Wang et al. reported nitromethane yields of 2.67 μg/cigarette [14] and Sampson et al. reported yields of 2.30 μg/cigarette [13] for an ISO regime. Sampson et al. reported nitromethane yields of 6.52 μg/cigarette for an HCI regime [13]. However, published studies of amounts of nitromethane in cigarette smoke generally do not identify the commercial products used as samples. Hoffmann et al. reported average nitromethane yields from 0.186 to 1.05 μg/cigarette (n = 200 cigarette/sample) in eight different products of unspecified origin [12]. In contrast, Wang et al. reported nitromethane yields in a range of 0.13–2.3 μg/cigarette for 10 different Chinese commercial products [14]; in both cases, product identities were unspecified. Potential reasons for the differences in 3R4F yields obtained using this method relative to the Sampson et al. method may include the use of peak area ratios for quantitation (rather than height) and differences in sampling techniques; potential explanations for differences in 3R4F yields relative to the Wang et al. method include the assessment of matrix effects as well as differences in sampling approaches, choice of internal standard, and number of cigarettes per sample.

## 4 Conclusions

Our new method provides a selective and fully validated technique to accurately quantify nitromethane in mainstream cigarette smoke. The method is sufficiently accurate and selective, exhibits minimal matrix effects and a low detection limit, and allows for high throughput sample collection and analysis. Even though machine-based smoking does not necessarily represent the high variability in individual smoking behaviors, this approach provides a reliable means for comparing deliveries across a wide range of products and smoking regimens.

## Disclaimer

The cigarette brands analyzed were obtained through a one-time purchase from a single product lot and do not necessarily reflect the lot-to-lot variability of nitromethane concentrations in these products over time. The findings and conclusions in this study are those of the authors and do not necessarily represent the official position of the Centers for Disease Control and Prevention. Use of trade names and commercial sources is for identification only and does not constitute endorsement by the U.S. Department of Health and Human Services or the Centers for Disease Control and Prevention.

## Funding

This project was supported in part by an appointment to the Research Participation Program for the Centers for Disease Control and Prevention, National Center for Environmental Health, Division of Laboratory Sciences (DLS), administered by the Oak Ridge Institute for Science and Education through an agreement between the U.S. Department of Energy and DLS.

## CRediT authorship contribution statement

**Juliana Giraldo Junco:** Methodology, Validation, Investigation, Writing - original draft. **Gala M. Chapman:** Conceptualization, Methodology, Validation, Investigation, Writing - review & editing. **Roberto Bravo Cardenas:** Investigation, Validation, Data curation, Writing - review & editing. **Clifford H. Watson:** Writing - review & editing, Supervision. **Liza Valentín-Blasini:** Writing - review & editing, Supervision.

## Declaration of Competing Interest

The authors report no declarations of interest.
